# Electrolyzed hypochlorous acid water exhibits potent disinfectant activity against various viruses through irreversible protein aggregation

**DOI:** 10.3389/fmicb.2023.1284274

**Published:** 2023-10-19

**Authors:** Rahmi Dianty, Junki Hirano, Itsuki Anzai, Yuta Kanai, Tsuyoshi Hayashi, Masae Morimoto, Chikako Kataoka-Nakamura, Sakura Kobayashi, Kentaro Uemura, Chikako Ono, Tokiko Watanabe, Takeshi Kobayashi, Kosuke Murakami, Kenji Kikuchi, Kunimoto Hotta, Toshikazu Yoshikawa, Shuhei Taguwa, Yoshiharu Matsuura

**Affiliations:** ^1^Laboratory of Virus Control, Center for Infectious Disease Education and Research, Osaka University, Osaka, Japan; ^2^Laboratory of Virus Control, Research Institute for Microbial Diseases, Osaka University, Osaka, Japan; ^3^Laboratory of Molecular Virology, Research Institute for Microbial Diseases, Osaka University, Osaka, Japan; ^4^Laboratory of Virology, Research Institute for Microbial Diseases, Osaka University, Osaka, Japan; ^5^Department of Virology II, National Institute of Infectious Diseases, Tokyo, Japan; ^6^Innovative Vaccine Research and Development Center, The Research Foundation for Microbial Diseases of Osaka University, Osaka, Japan; ^7^Center for Advanced Modalities and DDS, Osaka University, Osaka, Japan; ^8^Louis Pasteur Center for Medical Research, Kyoto, Japan

**Keywords:** hypochlorous acid, virucide, SARS-CoV-2, oxidation, protein aggregation

## Abstract

It is essential to employ efficient measures to prevent the transmission of pathogenic agents during a pandemic. One such method involves using hypochlorous acid (HClO) solution. The oxidative properties of HClO water (HAW) can contribute to its ability to eliminate viral particles. Here, we examined a highly purified slightly acidic hypochlorous acid water (Hp-SA-HAW) obtained from the reverse osmosis membrane treatment of an electrolytically-generated SA-HAW for its anti-viral activity and mode of action on viral proteins. Hp-SA-HAW exhibited broad-spectrum antiviral effects against various viruses, including adenovirus, hepatitis B virus, Japanese encephalitis virus (JEV), and rotavirus. Additionally, Hp-SA-HAW treatment dose-dependently resulted in irreversibly aggregated multimers of the JEV envelope and capsid proteins. However, Hp-SA-HAW treatment had no discernible effect on viral RNA, indicating that Hp-SA-HAW acts against amino acids rather than nucleic acids. Furthermore, Hp-SA-HAW substantially reduced the infectivity of severe acute respiratory syndrome coronavirus 2 (SARS-CoV-2), including the ancestral variant and other multiple variants. Hp-SA-HAW treatment induced the aggregation of the SARS-CoV-2 spike and nuclear proteins and disrupted the binding of the purified spike protein of SARS-CoV-2 to human ACE2. This study demonstrates that the broad-spectrum virucidal activity of highly purified HClO is attributed to viral protein aggregation of virion via protein oxidation.

## 1. Introduction

Hypochlorous acid (HClO) is a natural defense molecule produced by neutrophils as an oxidant through the myeloperoxidase pathway to eliminate many pathogens (Klebanoff, 1970; Winterbourn et al., 2006; Winterbourn, 2008). HClO water (HAW), defined as an aqueous solution containing HClO, is industrially produced by electrolysis reactions of chloride ions (Cl^−^) derived from low concentrations of NaCl or HCl. There are 3 types of HAWs based on their acidic pH range; strong (pH 2.2–2.7), weak (pH 2.7–5), and slight (pH 5–6.5; Yan et al., 2021). The dissociative equilibrium components of HClO (HClO, ClO^−^, and Cl_2_) in aqueous solutions vary in their existing forms depending on the pH of the solution, and they possess bactericidal and deodorizing properties (Fukuzaki, 2006; Wang et al., 2007). Due to its small molecular size and electrical neutrality, HClO can penetrate the cell wall and inner cell membrane and exert potent bactericidal action on the components, including nucleic acids in the cytoplasm, compared to ClO^−^ (Carr et al., 1997; Fukuzaki, 2006).

Chemically, HAW is commonly categorized as sodium hypochlorite (NaClO) solution, well known as bleaching agent. However, there are distinct differences between them. Firstly, HAW primarily consists of HClO, whereas NaClO solution contains ClO^−^ as the major component (John et al., 2013). Secondly, NaClO solutions have high alkalinity and can be obtained commercially with very high available chlorine concentrations (4%–6%). However, these high concentrations limit their use as they require significant dilutions, and they remain corrosive and irritating to metals and living organisms (National Center for Biotechnology Information, 2023). On the other hand, HAW is acidic and available in lower concentrations (80 mg/L or less), allowing for direct use without further dilution. Importantly, HAW, with its near-neutral pH, does not cause irritation to organs and mucous membranes (Pelgrift and Friedman, 2013).

The advantageous properties of HClO and HAW make them widely accepted for infection control in hospitals, including antibacterial intracanal irrigation, oral maxillofacial surgery, and mouthwash, to prevent nosocomial infections (Klebanoff, 1970; Winterbourn et al., 2006; Wang et al., 2007; Winterbourn, 2008; Block and Rowan, 2020). However, there is still room for improvement in these substances. One area that requires attention is the development of high-purity HAWs to accurately analyze the true properties of HClO itself. To address this, reverse osmosis treatment of HAWs was used to effectively remove coexisting Na + ions from the solution, resulting in the preparation of a highly purified slightly acidic hypochlorous acid water (Hp-SA-HAW), as detailed in section 2. *In vitro* experiments have demonstrated the effectiveness of HAW against various viruses (Park et al., 2007; Hakim et al., 2015; Miyaoka et al., 2021, 2023). However, the molecular mechanisms underlying the antiviral effects of HClO are still not fully understood. This study reveals that Hp-SA-HAW exhibits broad-spectrum disinfectant activity against DNA and RNA viruses, including both enveloped and non-enveloped viruses, in a dose-dependent manner. The antiviral effect is attributed to the irreversible aggregation of viral proteins, with little impact on genomic nucleotides.

## 2. Materials and methods

### 2.1. Cells

Huh7, BHK, and RD-A cells were obtained from JCRB cell bank (Osaka, Japan) and maintained in Dulbecco’s modified Eagle’s medium (DMEM; Nacalai Tesque, Inc., Kyoto, Japan) supplemented with 10% fetal bovine serum (FBS; Gibco, Gland Island, NY, United States), 100 units/mL penicillin, and 100 mg/mL streptomycin (Sigma-Aldrich, St. Louis, MO, United States). Additionally, BSR cells (Boyce et al., 2008), a derivative of BHK, and monkey kidney epithelial MA104 cells (kindly provided by Prof, Hiroshi Ushijima, Nihon University School of Medicine) were cultured in DMEM supplemented with 5% FBS. Chick embryo fibroblast (Kawagishi et al., 2016) and SBC3 (JCRB cell bank), a small cell lung carcinoma cell line, expressing ACE2/TMPRSS2 was cultured in Eagle’s minimum essential medium supplemented with 10% FBS, 100 units/mL penicillin, and 100 mg/mL streptomycin (Sigma-Aldrich). Moreover, the jejunal human intestinal organoid (HIO) J2 line was provided by Baylor College of Medicine under a material transfer agreement. To maintain HIO, the cells were embedded in Matrigel and cultured as 3D HIO in IntestiCult Organoid Growth Medium (STEMCELL, Vancouver, Canada; Ettayebi et al., 2016; Hayashi et al., 2021). Lastly, HepG2-hNTCP-C4 cells were maintained as previously described (Fauzyah et al., 2021). The study protocol was approved by the Review Board of the National Institute of Infectious Diseases in Japan.

### 2.2. Viruses

All viral data are presented in Table 1. All experiments involving severe acute respiratory syndrome coronavirus 2 (SARS-CoV-2) were performed in biosafety level-3 laboratories, and those involving the other viruses were conducted in biosafety level-2 laboratories, following the standard biosafety protocols approved by the Research Institute for Microbial Diseases at Osaka University. Japanese encephalitis virus (JEV) particles were filtered with 0.22 μm Millipore filter (Sigma-Aldrich) and concentrated with Vivaspin 100,000 MWCO centrifugal concentrator (Sartorius, Tokyo, Japan).

**Table 1:** Summary of viruses used in this study.

Family	Virus	Strain	Pango lineage	Cell
Flaviviridae	Japanese encephalitis virus	AT31		Huh7
Reoviridae	Rotavirus	SA11		MA104
Picornaviridae	Enterovirus A71	A71		RD-A
Caliciviridae	Human Norovirus	GII.4		Intestinal organoid
Hepadnaviridae	Hepatitis B virus	ayw		MRC-5
Adenoviridae	Adenovirus	type 5		Huh7
Poxviridae	Vaccinia virus	rDIs-T7pol		Chicken embryonic fibroblast
Coronaviridae	Severe acute respiratory syndrome coronavirus 2	2019-nCoV/Japan/TY/WK-521/2020	A-lineage	SBC3/ACE2/TMPRSS2
		hCoV-19/Japan/QHN001/2020	B1.1	
		hCoV-19/Japan/TY7-503/2021	P.1	
		hCoV-19/Japan/TY11-927/2021	AY.122	
		hCoV-19/Japan/TY38-873/2021	BA.1.1	

### 2.3. Virus-like particle preparation

The cDNA encoding P1 and 3CD of EV-A71 was inserted into the pcDNA3.4 vector. Next, both plasmids were co-transfected into ExpiCHO-S cells, according to the manufacturer’s instructions (Thermo Fisher Scientific, Waltham, MA, United States). At 6 days post-transfection, the supernatant was collected and centrifuged at 300 × *g* for 30 min and at 4°C to remove the debris. Afterward, the supernatant was processed using tangential filtration to concentrate the virus-like particles (VLPs). Subsequently, the concentrated VLPs were pelleted using ultracentrifugation at 100,000 × *g* for 4 h and at 4°C. Next, the pelleted VLPs were resuspended in phosphate-buffered saline [PBS(−)], and contaminants were removed by ammonium sulfate precipitation. Next, the VLP-containing supernatant was subjected to buffer exchange using PD-10 desalting columns (GE Life Sciences, Marlborough, MA, United States), followed by ultracentrifugation purification using a 10%–40% sucrose density gradient. Lastly, the VLP fractions were collected and concentrated using Amicon Ultra columns at 100,000 MWCO (Sigma-Aldrich).

### 2.4. Chemicals and antibodies

Antibodies against JEV capsid (Genetex, Irvine, CA, United States), E (Genetex), His-6 (Abcam, Cambridge, United Kingdom), and enterovirus 71 (EV71) viral protein 1 (VP1; Genetex) and VP2 (Merck) against SARS-CoV-2 N protein were gifted by Biomatrix Research Inc. (Chiba, Japan). Additionally, rabbit anti-NSP4 serum was raised against a synthetic SA11 NSP4 peptide spanning amino acid residues 158–171 (Eurofins Genomics, Tokyo, Japan). Lastly, TransIT®-mRNA Transfection Reagent was purchased from TAKARA Bio Inc. (Shiga, Japan).

### 2.5. Production and purification of Hp-SA-HAW

NaCl solution was electrolyzed using a diaphragm three-chamber electrolytic water generator (Nipro, Osaka, Japan; International application number: WO 2023/090447 Al). The products were subsequently subjected to a specific reverse osmosis membrane filtration to remove ions, such as Na^+^ ion (International application number: WO 2021/235554 Al). The pH, conductivity, and concentration of the original Hp-SA-HAW are listed in Table 2.

**Table 2:** Summary of the physical properties of highly purified slightly acidic hypochlorous acid water.

pH	6.3–6.7
Electric conductivity	10–20 μS cm^−1^
HClO	37–41 mg L^−1^
BrO_3_^−^	<0.001 mg L^−1^
Cl^−^	4–10 mg L^−1^
SO_4_^2−^	<0.01 mg L^−1^
Na^+^	2–5 mg L^−1^
NH_4_^+^	<0.01 mg L^−1^

### 2.6. Viral titration

Extracellular viruses were harvested by removing cell debris via centrifugation at 300 × *g* for 5 min and collecting the culture supernatant. For titration using the focus-forming assay, confluent competent cells were cultured in 48- or 96-well plates and inoculated with a limited 10-fold dilution series of the virus in a culture medium containing 2% FBS for 1 h. After removing the inoculum, the cells were overlaid with a medium supplemented with 0.8% methylcellulose and 2% FBS. After 1 day for rotavirus (RV), 2 days for SARS-CoV-2, and 3 days for JEV and green fluorescent protein (GFP)-expressing adenoviruses, the cells were fixed in 4% paraformaldehyde in PBS and permeabilized using 0.5% Triton X-100. Next, the infectious foci were stained using anti-NSP4 (RV), anti-N and S (SARS-CoV-2), or anti-E (JEV) antibodies and visualized using VECTASTAIN Elite ABC anti-mouse IgG kit with VIP substrate (Vector Laboratories, Newark, CA, United States). GFP-expressing adenoviruses were titrated by counting the GFP foci in the wells under a fluorescence microscope instead of staining with specific antibodies and VIP.

For titration of EV71 using plaque assay, confluent proficient cells were inoculated with a constrained 10-fold dilution series of the virus in a culture medium containing 2% FBS for 1 h. After removing the inoculum, the cells were coated with a medium enriched with 0.8% methylcellulose and 2% FBS. After 7 days, the cells were immobilized in 4% paraformaldehyde in PBS and stained using a 0.1% solution of methylene blue in PBS.

For the titration of Hepatitis B virus (HBV), MRC-5 cells were subjected to infection with a dosage of 10,000 genome equivalents [Geq] per cell. Subsequently, viral RNAs were extracted using the Purelink spin column kit by Thermo Fisher Scientific, followed by cDNA synthesis utilizing the High Capacity cDNA Reverse Transcription Kit from Life Technologies. qRT-PCR analysis was conducted with gene-specific primers (iTaqTM Universal Supermixes or SYBR-Green, Bio-Rad) following the respective manufacturer’s instructions. Quantification of the HBV genome was achieved via standard curve methods employing a DNA fragment encoding HBV as the calibration curve. To ensure consistency, each HBV quantity was normalized to the GAPDH housekeeping gene and expressed as arbitrary units representing HBV RNA.

Vaccinia virus titers were determined using the 50% tissue culture infectious dose (TCID_50_). Briefly, confluent monolayers of BSR cells in a 96-well plate were inoculated with a constrained 10-fold dilution series of the virus in a culture medium supplemented with 5% FBS and incubated for 1 h for viral adsorption. After removing the inoculum, the cells were cultured in DMEM supplemented with 5% FBS. At 72 h post-infection, the cells were fixed with 4% paraformaldehyde and stained with crystal violet to visualize CPE. The TCID_50_ of each virus was calculated as previously described.

### 2.7. Immunoblotting

The samples were lysed using RIPA buffer (25 mM Tris–HCl, 150 mM NaCl, 1% NP-40, 0.5% sodium deoxycholate, 0.1% sodium dodecyl sulfate [SDS]) or 6 M urea buffer (6 M urea, 150 mM NaCl, 20 mM Tris pH 8) supplemented with complete protease inhibitor cocktail (Sigma-Aldrich) for 30 min at 4°C, and then cells were triturated 10 times through a 25-gauge needle. After the determination of protein concentration by BCA assay, samples were adjusted with 1X SDS-sample buffer (50 mM Tris HCl pH 7.4, 5% SDS, 10% Glycerol) and subjected to SDS-polyacrylamide gel electrophoresis (SDS-PAGE). Immunoblot analysis was performed using primary antibodies as indicated and Li-cor secondary anti-mouse and anti-rabbit antibodies. The signals were assessed by fluorescent-based quantitative immunoblot analysis (Empiria Studio Software: Licor Odyssey co, Lincoln, NE, United States).

### 2.8. Protein oxidation assay

Viral protein modification by HAW oxidation reactions is mediated by new functional groups, such as carbonyl groups. Carbonyl proteins are formed via various oxidation mechanisms, indicating oxidative injury. The protein carbonyl count was determined by derivatizing with dinitrophenylhydrazine (DNPH) and measuring its binding to anti-DNPH antibodies (Immunodiagnostic AG, Bensheim, Germany). After the purified virus was prepared, the protein amount was determined using the BCA assay. Next, each 10 μL viral particle was treated with four doses of HAW (0, 1.2, 8, and 40 ppm), incubated, and centrifuged using an Amicon-ultra 100 MW. Each 4 μL sample was derivatized for 45 min and washed with 60 μL assay buffer in a centrifugal filtration concentrator four times. Subsequently, the standard, control, and sample were diluted at 1:100 in respective wells and incubated overnight at 4°C. Next, the anti-DNPH antibody was manually added. Lastly, the protein carbonyl content of the treated and control groups was determined using Glomaxâ Discovery Microplate Reader (Promega, Madison, WI, United States) at 450 nm and calculated using a reference diluted standard of the protein carbonyl assay.

### 2.9. RNA oxidation assay

RNA was extracted from the supernatants of JEV-infected cells using a PureLink RNA Mini Kit (Invitrogen, Waltham, MA, United States) and digested to nucleosides using 20 units of nuclease P1 for 2 h at 37°C in 10 mM sodium acetate and 10 units of alkaline phosphatase for 1 h at 37°C in 100 mM Tris, pH 7.5 (New England BioLabs Inc., Ipswich, MA, United States). Lastly, RNA oxidation was evaluated by quantitative measurement of 8-hydroxyguanosine (8-OHG; Cayman Chemical Company, Ann Arbor, MI, United States) according to the manufacturer’s protocol.

### 2.10. RBD-ACE binding assay

Microtiter plate wells were coated with 1 μg/mL spike protein (D614G) in 50 μL carbonate–bicarbonate buffer (1.59 g Na_2_CO_3_ and 2.93 g NaHCO_3_ dissolved in 1 L deionized water, pH 9.6, 0.4 pmol). After incubating the plate at 4°C overnight, 100 μL of diluted HAW with PBS at various concentrations (40.0–0.625 ppm) was added, incubated for 30 min, and washed with PBS. The reaction was blocked with 5 mg/mL bovine serum albumin (BSA) in 200 μL PBS at 4°C overnight. Next, 1 μg/mL hACE2 diluted in 100 μL PBS was added and incubated for 2 h. After washing with PBS, the test and control samples were reacted with a goat anti-rabbit IgG antibody, horseradish peroxidase-conjugate (Jackson Laboratory, Bar Harbor, ME, United States) at a dilution of 1:10,000 in PBS. After 2 h of incubation at 25°C with the appropriate secondary antibody substrate solution and 0.4 mg/mL OPD/0.03% H_2_O_2_ in citrate buffer, pH 5, absorbance was measured at 492 nm.

### 2.11. Evaluation of the effect of Hp-SA-HAW on human norovirus infection in HIO

To conduct the HuNoV infection experiment using the HIO culture system, differentiated 2D HIO monolayers were prepared as described previously (Ettayebi et al., 2016; Hayashi et al., 2021). Briefly, 3D HIOs were dissociated with TrypLE Express (Thermo Fisher Scientific) and seeded onto 96-well plates pre-coated with collagen IV at a density of approximately 10^5^ cells/well in IntestiCult Organoid Growth Medium containing the ROCK inhibitor Y-27632 (10 μM, Sigma-Aldrich) for 2–3 days. Next, the medium was replaced with IntestiCult Organoid Differentiation Medium (IntestiCult ODN, STEMCELL), and the cells were maintained for 2 days. GII.4 [P16] HuNoV, containing 8.55 × 10^5^ genome equivalents in 25 μL of 10% stool filtrate, was treated with an equal volume of PBS as a non-treatment control or 0.12, 0.6, 3, or 15 ppm of HAW for 30 min at 25°C. Next, one 100 μL of IntestiCult ODN containing 3% FBS and 750 μM GCDCA were added to the samples. Subsequently, the 2D HIO monolayers in 96-well plates were inoculated with 100 μL of the above mixtures for 1 h. Next, the cells were washed twice with a complete medium without growth factors (−) and cultured in IntestiCult ODN in the presence of 500 μM GCDCA, which promotes GII.4 HuNoV infection (Murakami et al., 2020). After 24 h post-infection, the cells and medium were harvested and subjected to RNA extraction using a Direct-zol-96 RNA Kit (Zymo Research, Irvine, CA, United States). Lastly, the extracted RNAs were subjected to reverse transcription-quantitative polymerase chain reaction to measure HuNoV GEs using TaqMan Fast Virus 1-Step Master Mix (Thermo Fisher Scientific) and GII-specific primer/probe sets (Kageyama et al., 2003; Hayashi et al., 2021).

### 2.12. Quantification and statistical analysis

Data plotting and statistical analyses were performed using Prism 9.3.1 (GraphPad Software, San Diego, CA, United States). Details on the number of technical and biological (independent) replicate for each experiment can be found in the figure legends. Each significance level described in the figure legends is indicated by an asterisk (*). Statistical significance was set at *p* < 0.05.

## 3. Results

### 3.1. Hp-SA-HAW exhibits potent disinfectant activity against various viruses

**Figure 1:**
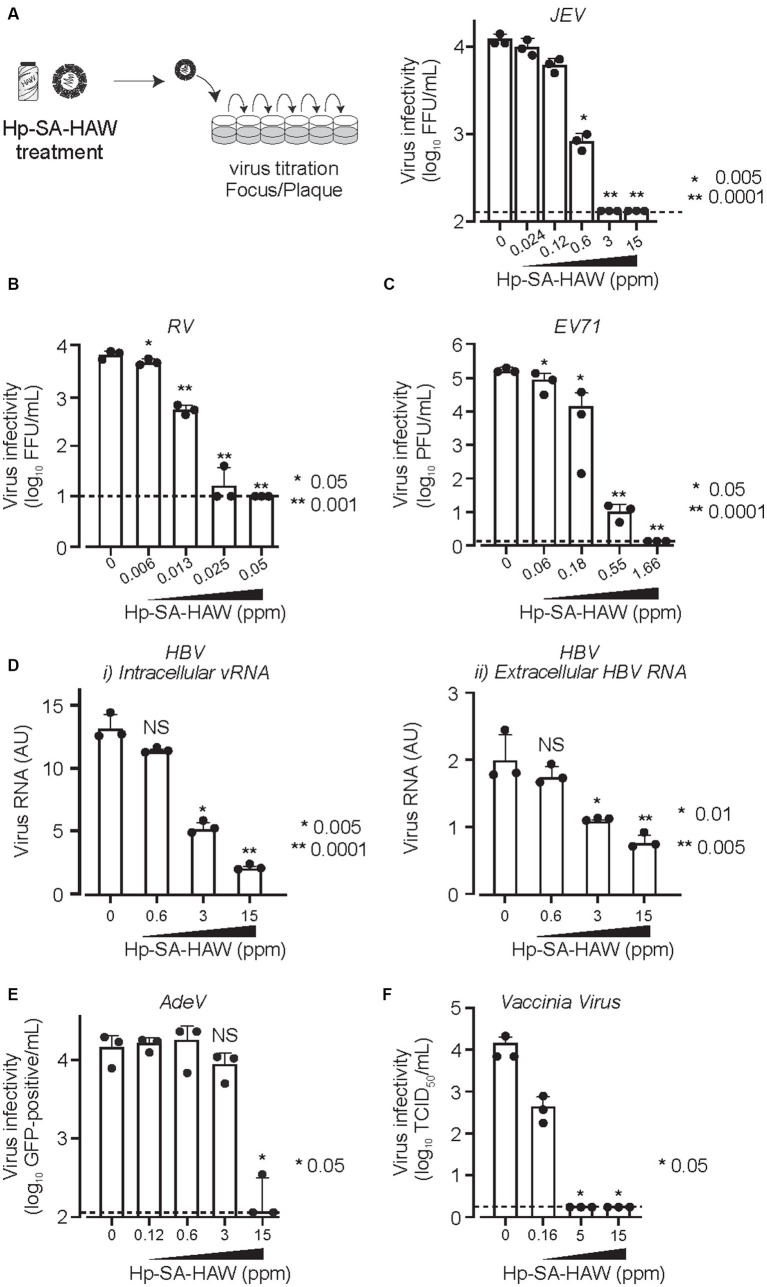
Hp-SA-HAW demonstrates significant virucidal efficacy against various viruses. **(A)** Japanese encephalitis virus (JEV, 10^4^ FFU) was incubated with 0.024, 0.12, 0.6, 3, and 15 ppm Hp-SA-HAW for 30 min at room temperature. The reaction was terminated by adding 2% FBS. The virus titers were determined following the procedures described in section 2. **(B)** Rotavirus (RV, 10^4^ PFU) was exposed to 0.006, 0.013, 0.025, and 0.05 ppm Hp-SA-HAW. **(C)** Enterovirus 71 (EV71, 10^5^ PFU) was treated with 0.06, 0.18, 0.55, and 1.66 ppm Hp-SA-HAW. **(D)** Hepatitis B virus (HBV) genotype D (10,000 genome equivalents [Geq]/cell) was treated with 0.6, 3, and 15 ppm Hp-SA-HAW. Intracellular **(i)** and extracellular viral RNAs **(ii)** were collected and evaluated using qRT-PCR and showed as the arbitral unit (AU) of HBV RNA. **(E)** Adenovirus type 5 expressing GPF (10,000 GFP foci/cells) was treated with 0.12, 0.6, 3, and 15 ppm Hp-SA-HAW. **(F)** Vaccinia virus (10^4^ PFU) was treated with 0.16, 5, and 15 ppm Hp-SA-HAW. The data represent the average of triplicate experiments. Error bars represent the standard deviation of the mean, and asterisks indicate significant differences between treated and non-treated groups determined using the One-way ANOVA with a Dunnett’s multiple comparisons test for all figures.

We confirmed the antiviral activity of Hp-SA-HAW (pH 5.0–6.5) against various DNA or RNA and enveloped or non-enveloped viruses (Table 1). Consistent with previous studies on HClO solution, Hp-SA-HAW treatment significantly reduced the infectivity of the JEV, an enveloped RNA virus, dose-dependently (Figure 1A). The viral titer decreased at 0.6 ppm and completely disappeared at over 3 ppm, reducing by >2 logs. Additionally, Hp-SA-HAW was more effective against non-enveloped RNA viruses, such as RV and EV71, than against JEV (Figures 1B,C). Virucidal effects against RV and EV71 appeared at 0.006 and 0.06 ppm, and 4 logs reduction was observed at 0.05 and 1.66 ppm of Hp-SA-HAW, respectively. Furthermore, we examined the effects of Hp-SA-HAW on DNA viruses. Hp-SA-HAW was less effective against hepatitis B virus (HBV) than against RNA viruses (Figure 1D). Specifically, HBV underwent intracellular replication (Figure 1Di) with 15 ppm Hp-SA-HAW; consequently, Hp-SA-HAW was less effective against the extracellular titer of HBV at 15 ppm (Figure 1Dii). However, 15 ppm Hp-SA-HAW treatment resulted in 2 logs reduction in adenovirus (Figure 1E) and vaccinia virus (Figure 1F).

### 3.2. Hp-SA-HAW oxidizes viral proteins and induces viral protein aggregation

**Figure 2:**
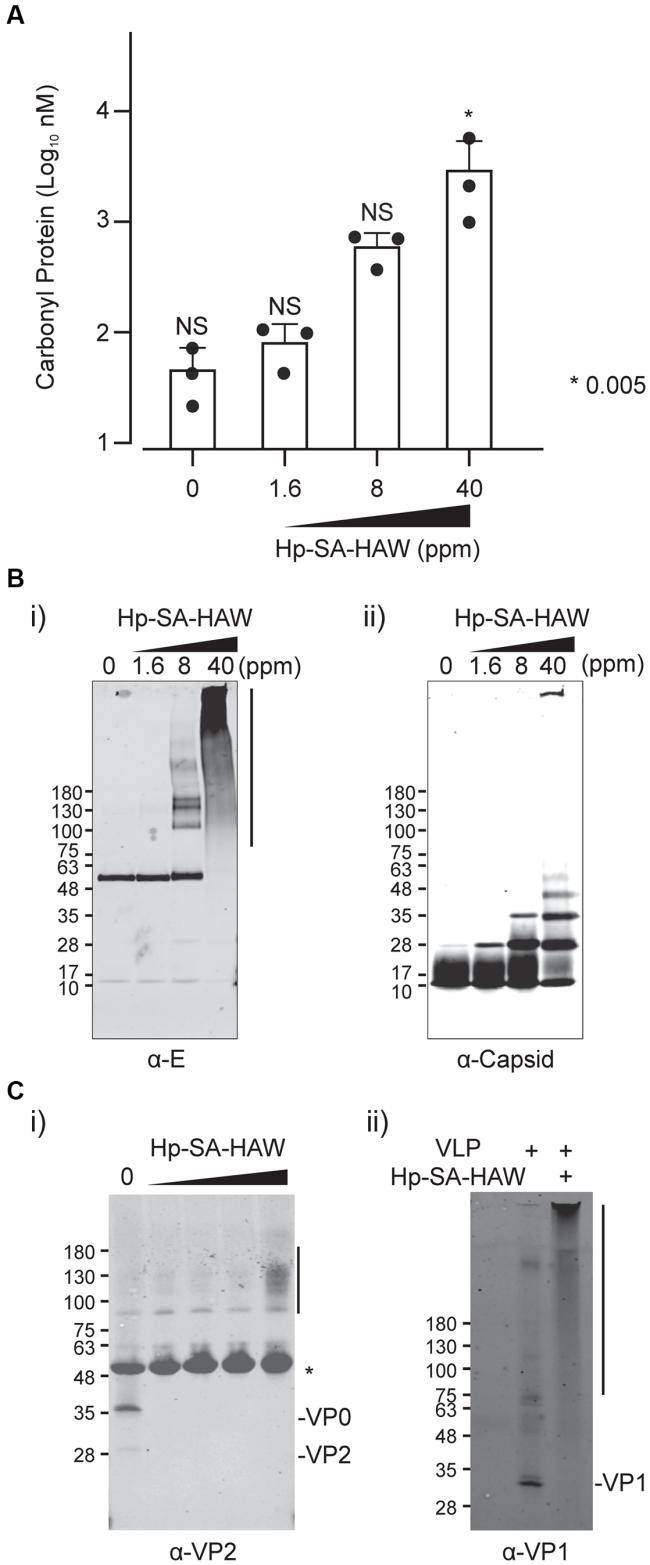
Hp-SA-HAW mediated oxidation of viral proteins and formation of viral protein aggregates. **(A)** Japanese encephalitis virus (JEV) was exposed to 1.6, 8, and 40 ppm Hp-SA-HAW. The levels of carbonylated viral proteins were quantified using an ELISA kit, following the protocols outlined in section 2. **(B)** Hp-SA-HAW-treated JEV samples were subjected to SDS-PAGE, followed by immunoblotting using antibodies specific to JEV envelope (E) protein **(i)** and capsid protein **(ii)**. **(C)** Enterovirus 71 (EV71) VLP exposed to Hp-SA-HAW was subjected to SDS-PAGE, followed by immunoblotting using antibodies targeting EV71 viral protein 2 (VP2) **(i)** and viral protein 1 (VP1) **(ii)**. The data represent the average of triplicate experiments. Error bars represent the standard deviation of the mean, and asterisks indicate significant differences between treated and non-treated groups determined using the One-way ANOVA with a Dunnett’s multiple comparisons test for all figures.

Since HClO is a potent oxidant, we assumed that the viral particle components were oxidized following Hp-SA-HAW treatment. To investigate the mechanisms underlying the antiviral action of Hp-SA-HAW, we first determined the oxidation of viral proteins. Oxidated amino acids form protein carbonyls; therefore, we measured the amount of carbonylated viral particles following Hp-SA-HAW treatment using enzyme-linked immunosorbent assay (ELISA). JEV proteins concentrated using ultrafiltration were significantly oxidized upon treatment with 40 ppm Hp-SA-HAW (Figure 2A). Furthermore, protein oxidation induces changes in protein structure triggered by non-specific interactions and produces high-molecular-weight protein aggregates or fragments of the protein backbone by degradation (Hawkins and Davies, 1998; Hawkins et al., 2003), demonstrated by the increased molecular weight and disappearance of protein bands in SDS-PAGE, respectively. The JEV capsid binds to the viral RNA and is enveloped by the matrix (M) and envelope proteins (E) to form viral particles. The JEV E protein showed concentration-dependent protein multimerization (slightly at 1.6 ppm and completely at 8 ppm); specifically, smears of high molecular weight species were detected at high concentrations of Hp-SA-HAW (8 and 40 ppm; Figure 2Bi). Additionally, the capsid protein formed a dimer under Hp-SA-HAW treatment at 1.6 ppm (Figure 2Bii). As the concentration increased, the complex formed multimeric pairs, and high-molecular-weight protein aggregates were detected at the top of the SDS-PAGE at 40 ppm Hp-SA-HAW, suggesting that Hp-SA-HAW treatment induced viral protein aggregation. Furthermore, virion component degeneration was observed in non-enveloped EV71 VLPs (Figure 2C). The EV71 capsid protein, viral protein (VP0) and its processed product VP2 disappeared after Hp-SA-HAW treatment at mild concentrations, suggesting capsid degradation (Figure 2Ci). Additionally, smears of the aggregated viral protein were observed at high concentrations of Hp-SA-HAW. A similar smear of VP1 aggregates was observed (Figure 2Cii) These protein aggregates were detergent-insoluble, as harsh detergent treatments, such as RIPA buffer (SDS, triton, and deoxycholate) or 6 M urea solution, were unable to dissolve the smear of protein aggregates (Supplementary Figures 1A,B). Overall, these results suggest that Hp-SA-HAW oxidizes virion components, resulting in irreversible protein aggregation.

### 3.3. Viral RNAs in virion are resistant to Hp-SA-HAW treatment

**Figure 3:**
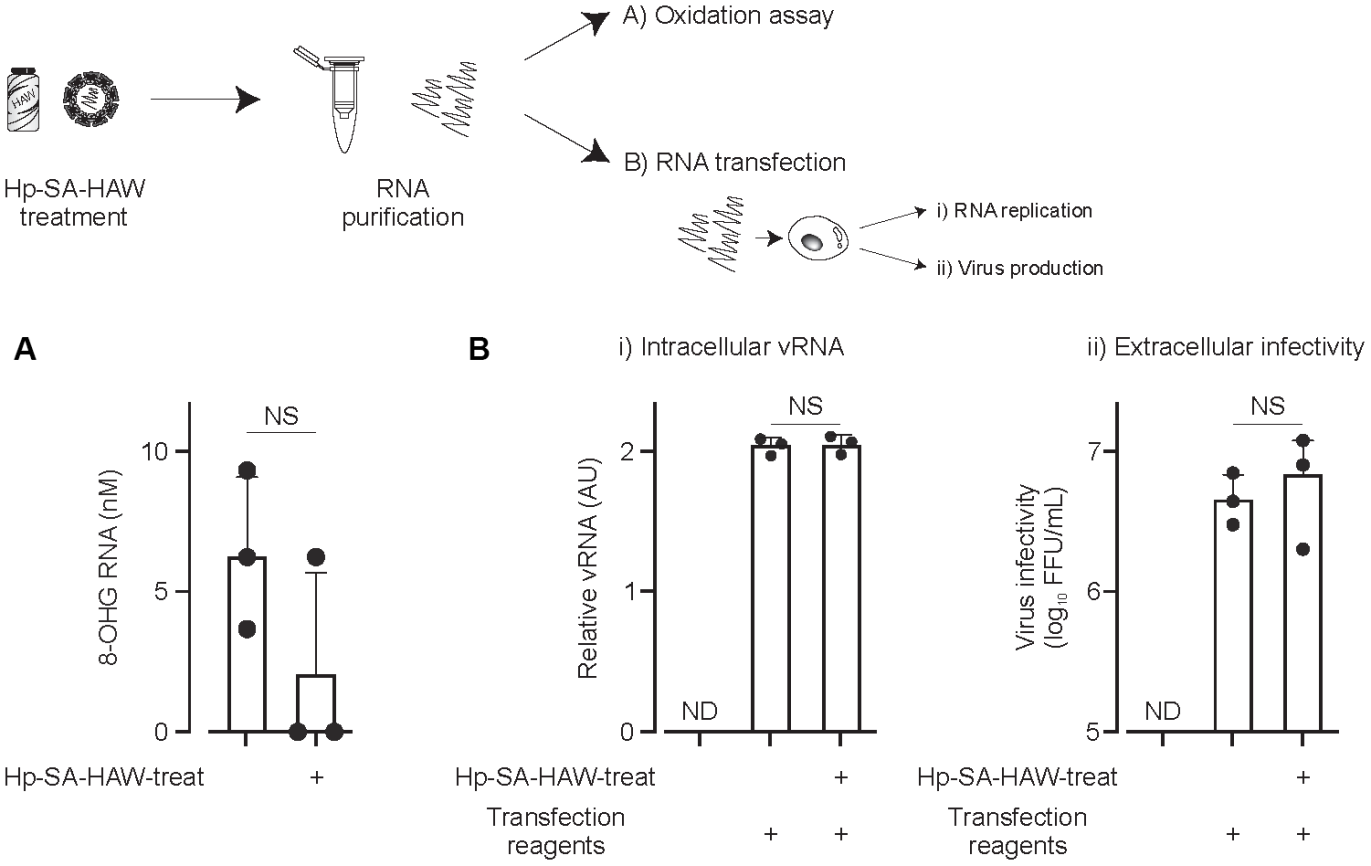
Viral RNAs within the virions resist Hp-SA-HAW treatment. **(A)** Japanese encephalitis virus (JEV) was exposed to 40 ppm Hp-SA-HAW. The viral RNA was subsequently purified, and 8-hydroxyguanosine levels were quantified using an ELISA kit. The quantification was performed following the established protocols described in section 2. **(B)** JEV RNA isolated from virions treated with Hp-SA-HAW was transfected into Huh7 cells using the TransIT-mRNA Transfection Kit. The intracellular viral RNA **(i)** and extracellular virus titer **(ii)** were determined 3 days after transfection through qRT-PCR and focus-forming assay, respectively. The data represent the average of triplicate experiments. Error bars represent the standard deviation of the mean and the significant differences determined using the Student’s *t*-test for all figures.

We investigated the effects of Hp-SA-HAW on viral RNA. Notably, 8-OHG is an oxidatively damaged guanine and a measurable marker of oxidation. JEV virions in the culture medium were treated with Hp-SA-HAW, and the RNA extracted from the viral particles was subjected to competitive ELISA for the quantitative measurement of 8-OHG. We observed no differences in the degree of RNA oxidation between viral particles with or without Hp-SA-HAW treatment (Figure 3A). Additionally, the viral RNA purified from JEV particles with or without Hp-SA-HAW exhibited compatible intracellular viral RNA (Figure 3Bi) and extracellular infectivity (Figure 3Bii) upon transfection into Huh7 cells. These results suggest that the genomic RNA in the viral particles is resistant to Hp-SA-HAW treatment, and the antiviral effect of Hp-SA-HAW is primarily due to the denaturation of viral proteins rather than viral RNA.

### 3.4. Hp-SA-HAW exhibits a potent disinfectant activity against SARS-CoV-2

**Figure 4:**
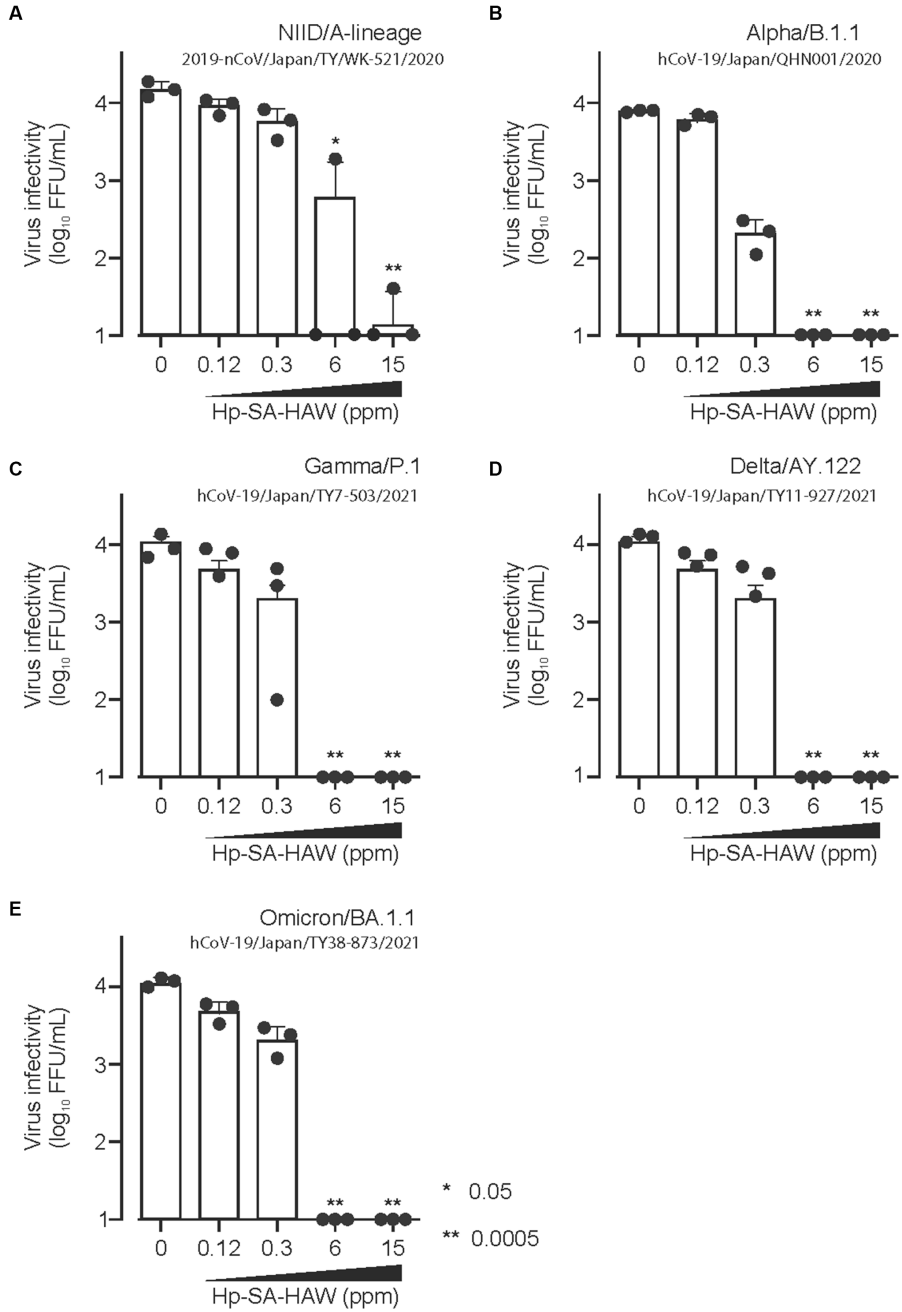
Hp-SA-HAW exhibits remarkable efficacy as a disinfectant against SARS-CoV-2, the etiological agent of coronavirus disease. **(A–E)** Each variant of SARS-CoV-2 was exposed to 0.12, 0.3, 6, and 15 ppm Hp-SA-HAW for 30 min at room temperature. The reaction was halted by adding 2% FBS. Subsequently, the virus titers were determined using the procedures outlined in section 2. The data represent the average of triplicate experiments. The error bars correspond to the standard deviation of the mean, and asterisks denote significant differences between treated and non-treated groups determined using the One-way ANOVA with a Dunnett’s multiple comparisons test for all figures.

**Figure 5:**
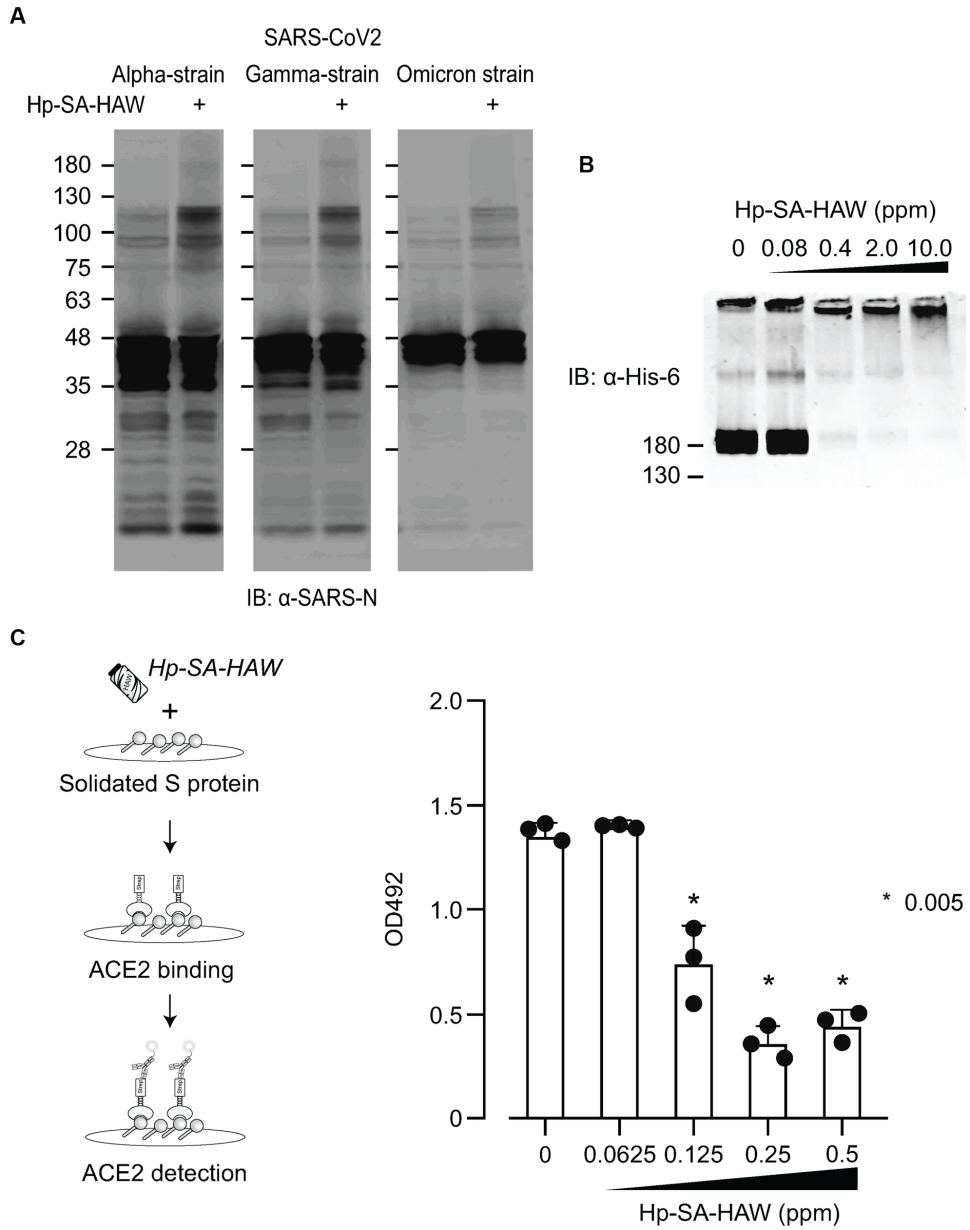
Hp-SA-HAW treatment induces aggregation of S proteins, reducing their receptor binding capacity. **(A)** The concentrated three SARS-CoV-2 variants were exposed to 40 ppm Hp-SA-HAW. The reaction was terminated by adding 2% FBS, and the samples were subjected to SDS-PAGE. Immunoblotting was performed using antibodies specific to the SARS-CoV-2 N protein. **(B)** Recombinant SARS-CoV-2 spike protein with a His tag was treated with Hp-SA-HAW and analyzed using immunoblotting with a His-6 antibody. **(C)** The solidified S protein was treated with HAW, and the binding of the S protein to recombinant strep-tagged ACE2 was examined following the procedures in section 2. The data represent the average of triplicate experiments. Error bars represent the standard deviation of the mean and the significant differences between treated and non-treated groups determined using the One-way ANOVA with a Dunnett’s multiple comparisons test for all figures.

Considering the ongoing pandemic, we assessed the virucidal efficacy of Hp-SA-HAW against SARS-CoV-2. Hp-SA-HAW considerably reduced the infectivity of SARS-CoV-2, specifically the 2019-nCoV/Japan/TY/WK-521/2020 variant prevalent during the early phase of the pandemic, dose-dependently (Figure 4A). Additionally, Hp-SA-HAW displayed substantial antiviral effect against various SARS-CoV-2 variants, including alpha, gamma, delta, and omicron variants, which can evade neutralizing antibodies elicited by mRNA vaccines (Figures 4A–E) Furthermore, we investigated the state of the virion components after Hp-SA-HAW treatment to explore the mechanism by which Hp-SA-HAW exhibits virucidal activity against SARS-CoV-2 (Figure 5). Similar to JEV and EV71, the nucleocapsid protein band (46 kDa) shifted to approximately 100–120 kDa after Hp-SA-HAW treatment (Figure 5A). Moreover, we analyzed the purified S protein of SARS-CoV-2 after Hp-SA-HAW treatment using immunoblotting (Figure 5B). Hp-SA-HAW treatment shifted the S protein from 180 kDa to just below the top of the well, and the extent of this shift increased dose-dependently, indicating that Hp-SA-HAW induced aggregation of the SARS-CoV-2 S protein. Lastly, to examine Hp-SA-HAW effect on the binding activity of the S protein to human ACE2, an entry receptor of SARS-CoV-2, we incubated the purified S protein solidified on a plate with human ACE2 after Hp-SA-HAW treatment and quantified the amount of bound hACE2 using ELISA (Figure 5C). Consistent with the immunoblotting results, Hp-SA-HAW treatment at >0.125 ppm significantly inhibited S protein binding to human ACE2 (Figure 5C). These results suggest that Hp-SA-HAW treatment induced viral protein aggregation by inhibiting the interaction between the S protein and human ACE2, leading to potent disinfectant activity against SARS-CoV-2.

### 3.5. Hp-SA-HAW inhibits the replication of human norovirus in HIO

**Figure 6:**
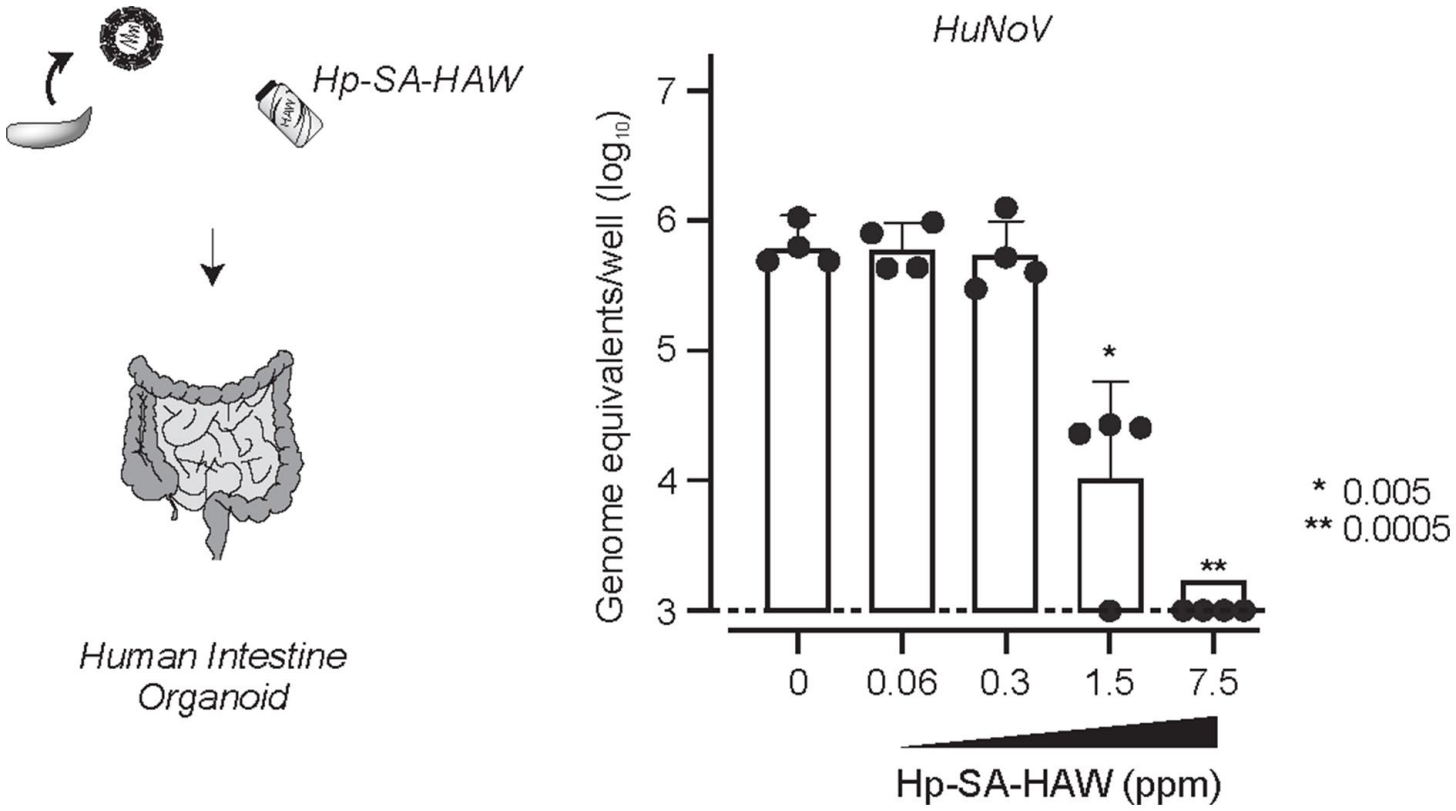
Hp-SA-HAW inhibits the replication of HuNoV in human intestinal organoid. HuNoV-containing stool filtrates were exposed to 0.06, 0.3, 1.5, and 7.5 ppm Hp-SA-HAW for 30 min at room temperature. The reaction was halted by adding 2% fetal bovine serum (FBS). Subsequently, virus copy numbers were determined using the procedures outlined in section 2. The data represent the average of four separate experiments. The error bars correspond to the standard deviation of the mean, and asterisks denote significant differences between treated and non-treated groups determined using the One-way ANOVA with a Dunnett’s multiple comparisons test for all figures.

Despite the broad-spectrum antiviral effects of Hp-SA-HAW, 2% FBS reduced its activity (Supplementary Figure 2), consistent with a previous study (Ishihara et al., 2017), suggesting that excess organics hinders the antiviral activity of Hp-SA-HAW. Therefore, we investigated the potential of Hp-SA-HAW as a disinfectant for HuNoV prepared from feces (Figure 6). The effectiveness of Hp-SA-HAW in reducing the viral infectivity of the HuNoV isolate was assessed using the HIO system. Treatment with 1.5 ppm Hp-SA-HAW reduced the viral titer, and complete viral inactivation was achieved at 7.5 ppm without any signs of cytotoxicity. These results suggest that Hp-SA-HAW can effectively neutralize HuNoV, even in the presence of impurities.

## 4. Discussion

Several studies have reported the mechanisms of disinfection by HAW, which are as follows: (1) Oxidation: HClO penetrates the cell membrane and disrupts the cells by oxidizing lipids and proteins, thereby inactivating pathogens (Slivka et al., 1980; Clark and Szot, 1981; McKenna and Davies, 1988; Hazen et al., 1998). (2) Damage to enzymes: HClO inactivates enzymes of microorganisms essential for energy production and cell replication necessary for survival (Knox et al., 1948; Hawkins and Davies, 2005). (3) DNA damage: HClO induces damage to the genetic material of the microorganisms by crosslinking nucleic acids, disrupting the replication, and leading to cell death (Albrich et al., 1981; Suquet et al., 2010). However, proving the direct antiviral effect of HClO is difficult because the hypochlorite water used in previous experiments also contains many impurities. In this study, we utilized a novel procedure to generate a highly purified HClO solution and demonstrated that Hp-SA-HAW treatment induced the oxidation and aggregation of viral proteins. The oxidation of viral proteins was discernible above the 1.6 ppm threshold, and a reduction in infectious titer and protein aggregation was observable at approximately 0.6 ppm, implying that protein aggregation alone is adequate for viral inactivation. Nucleic acids are an intriguing potential target for HClO treatment (Hawkins and Davies, 2002; Jiang et al., 2003), whereby nucleic chloramine formation produces mutagenic, genotoxic, and cytotoxic effects (Bernofsky, 1991; Tantry et al., 2018). Notably, no oxidation was observed in the viral RNA of the purified viral particles treated with Hp-SA-HAW (Figure 3A), and the purified viral RNA from the particles treated with Hp-SA-HAW displayed compatible infectivity following transfection into cells (Figure 3B). These results suggest that the antiviral effect of Hp-SA-HAW is predominantly due to the denaturation of viral proteins rather than viral RNA. Moreover, although HClO can disrupt lipid membranes, non-enveloped viruses, including EV71, RV, and norovirus, exhibited greater susceptibility to Hp-SA-HAW than enveloped viruses. This suggests that amino acids, rather than nucleic acids or lipid membranes, are the essential targets for Hp-SA-HAW’s antiviral activity.

HClO solutions demonstrate potent and broad-spectrum antiviral activity with minimal damage to mucous membranes and internal organs and is proposed as an alternative disinfectant to prevent infections that frequently occur in hospitals, particularly during invasive procedures, such as endoscopy and otolaryngology surgery (Overholt et al., 2018; Jiang and Liang, 2022; Okano et al., 2022). As reported previously, the efficacy of HClO solution as a bactericide and virucide is reduced by organic substances, such as BSA and FBS (Wang et al., 2007; Hakim et al., 2015). In a previous study, 3% BSA attenuated the bactericidal effect of chlorhexidine gluconate, benzethonium chloride, benzalkonium chloride, and alkyl diaminoethyl glycine hydrochloride, which are low-susceptibility disinfectants (Kawamura-Sato et al., 2008). Docking analysis was used to demonstrate this mechanism of attenuation, showing that organic compounds interact with HClO to reduce the chlorinating activity of the fungicides (Kumorkiewicz-Jamro et al., 2020). Consistent with other HClO solution studies, the antiviral activity of Hp-SA-HAW was lost in the presence of 2% FBS (Supplementary Figure 2). Nevertheless, treatment with high concentrations of Hp-SA-HAW completely eradicated HuNoV prepared from feces (Figure 6). In the case of the hepatitis B virus (HBV), it exhibited higher resistance to Hp-SA-HAW, retaining its infectivity even when subjected to concentrations as high as 15 ppm, a level at which other viruses typically lose their infectivity. Notably, HBV has been found to release an astonishing 1,000 to 10,000 times more non-infectious particles than infectious ones (Blumberg, 1977; Ganem and Prince, 2004). These non-infectious particles could serve a crucial role in the virus’s evasion of the host immune system (Hu and Liu, 2017), and they may also function as a protective shield against inactivating agents. Further investigations are required to establish the clinical application of Hp-SA-HAW as a disinfectant.

In a previous report, modified albumin activated by HClO reacted with the envelope domain III of the West Nile virus and reduced its infectivity by 53% (Vossmann et al., 2008). Treatment of the purified S protein of SARS-CoV-2 with Hp-SA-HAW induced aggregation in a cell-free system (Figure 5B). The receptor binding site of the S protein contains several methionine and cysteine residues, which are highly reactive to HClO (Villamena, 2017). In our study, all the SARS-CoV-2 variants investigated, which have enhanced affinity to the receptor, exhibited similar aggregation following Hp-SA-HAW treatment, suggesting that Hp-SA-HAW will be effective against emerging variants. The ancestral Wuhan strain appears to be more resistant to Hp-SA-HAW than other mutant strains. While VOC has the advantage of improved cleavage efficiency leading to membrane fusion activation and immune evasion from neutralizing antibodies (Mlcochova et al., 2021; Peacock et al., 2021; Carabelli et al., 2023), structural changes may make it susceptible to chemical reactions such as hypochlorous acid. Further analysis with structural biology in the future will develop the discussion from a viral evolutionary perspective. To prepare for the next pandemic caused by airborne viruses, such as SARS-CoV-2 and influenza viruses, antiviral substances that inactivate viral proteins are crucial for infecting susceptible cells. This is the first study to show that Hp-SA-HAW directly aggregates viral proteins and inactivates viral infectivity.

In conclusion, the promising attributes of HClO as a potential antiviral agent for airborne and droplet-transmitted pathogens, coupled with its cost-effectiveness and absence of observed metal corrosion or chemical harm during environmental application, suggest its significance as a valuable agent in alleviating the global pandemic impact.

## Data availability statement

The raw data supporting the conclusions of this article will be made available by the authors, without undue reservation.

## Author contributions

RD: Conceptualization, Investigation, Project administration, Writing – review & editing. JH: Data curation, Investigation, Project administration, Writing – review & editing. IA: Investigation, Project administration, Writing – review & editing, Methodology. YK: Investigation, Project administration, Writing – review & editing, Methodology. TH: Investigation, Methodology, Project administration, Writing – review & editing. MM: Investigation, Project administration, Writing – review & editing. CK-N: Investigation, Project administration, Writing – review & editing, Methodology. SK: Investigation, Project administration, Writing – review & editing. KU: Investigation, Project administration, Writing – review & editing. CO: Investigation, Project administration, Writing – review & editing. TW: Project administration, Writing – review & editing. TK: Project administration, Writing – review & editing. KM: Project administration, Writing – review & editing. KK: Investigation, Project administration, Writing – review & editing. KH: Project administration, Writing – review & editing. TY: Project administration, Writing – review & editing. ST: Conceptualization, Funding acquisition, Investigation, Project administration, Supervision, Writing – original draft, Writing – review & editing. YM: Conceptualization, Funding acquisition, Project administration, Supervision, Writing – original draft, Writing – review & editing.

## Abbreviations

EV71: Enterovirus 71
ELISA: Enzyme-linked immunosorbent assay
HAW: HClO water
HBV: Hepatitis B virus
HClO: Hypochlorous acid
Hp-SA-HAW: Highly purified slightly acidic hypochlorous acid water
HIO: Human intestinal organoid
HuNoV: Human norovirus
GFP: Green fluorescent protein
JEV: Japanese encephalitis virus
SARS-CoV-2: Severe acute respiratory syndrome coronavirus 2
NaClO: Sodium hypochlorite
VLP: Virus-like particle
VP: Viral protein
RV: Rotavirus
SDS: Sodium dodecyl sulfate
SDS-PAGE: Sodium dodecyl sulfate-polyacrylamide gel electrophoresis
