# Supplementary figures and images for: Electrolyzed hypochlorous acid water exhibits potent disinfectant activity against various viruses through irreversible protein aggregation
Source: Front Microbiol. 2023 Oct 19;14:1284274. doi: 10.3389/fmicb.2023.1284274 (PMC10625411; doi:10.3389/fmicb.2023.1284274)

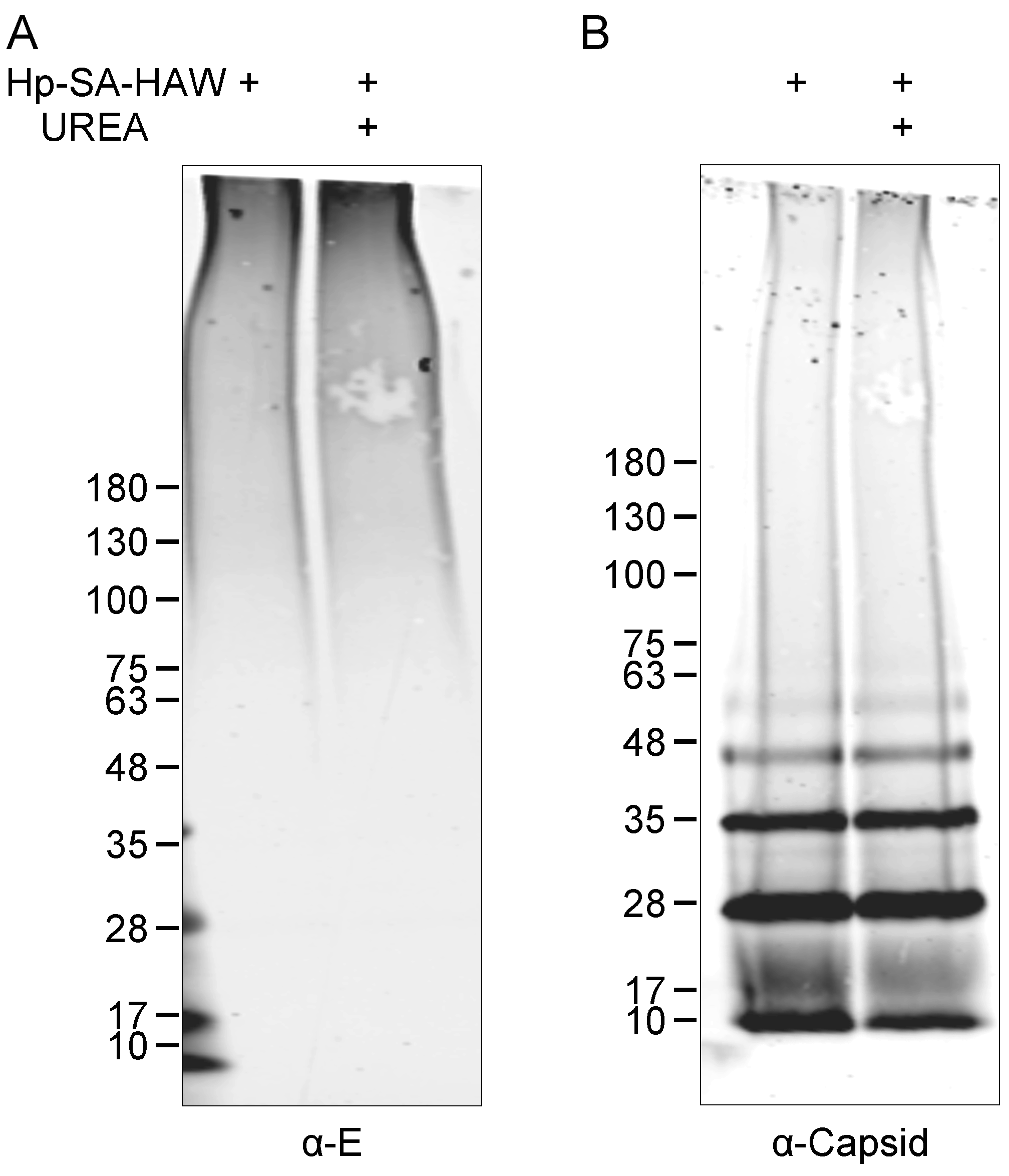

Supplement: Supplementary file 1 [file Image_1.TIFF]

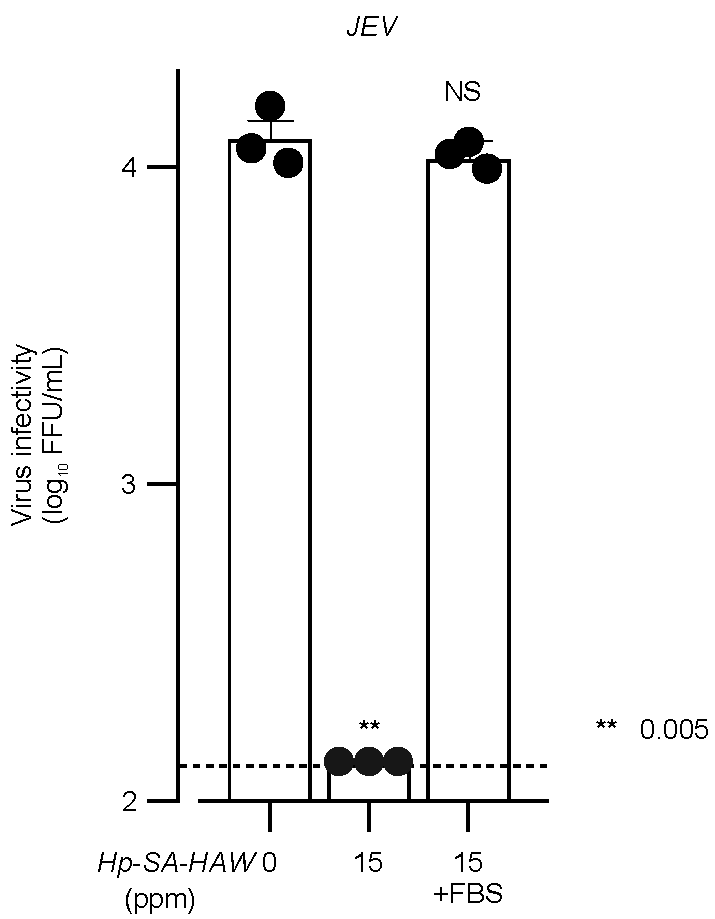

Supplement: Supplementary file 2 [file Image_2.TIFF]
